# Predictors of Pregnancy after Artificial Insemination in Women with Polycystic Ovary Syndrome

**DOI:** 10.5935/1518-0557.20240095

**Published:** 2025

**Authors:** Tânia Moreira, Carla Leal, Márcia Barreiro, António Tomé, Emídio Vale-Fernandes

**Affiliations:** 1 ICBAS - School of Medicine and Biomedical Sciences, UMIB - Unit for Multidisciplinary Research in Biomedicine, University of Porto, Porto, Portugal; 2 Centro de Procriação Medicamente Assistida / Banco Público de Gâmetas, Centro Materno-Infantil do Norte Dr. Albino Aroso (CMIN), Centro Hospitalar Universitário de Santo António (CHUdSA), Unidade Local de Saúde de Santo António (ULSSA), Porto, Portugal; 3 Departamento da Mulher e da Medicina Reprodutiva, Centro Materno-Infantil do Norte Dr. Albino Aroso (CMIN), Centro Hospitalar Universitário de Santo António (CHUdSA), Unidade Local de Saúde de Santo António (ULSSA), Porto, Portugal; 4 ITR - Laboratory for Integrative and Translational Research in Population Health, University of Porto, Porto, Portugal

**Keywords:** polycystic ovary syndrome, artificial insemination, intrauterine insemination, anti-Müllerian hormone, body mass index, reproductive outcomes

## Abstract

**Objective:**

Polycystic Ovary Syndrome (PCOS) is the most common endocrine disorder in women of reproductive age, being one of the main causes of infertility. Anti-Müllerian hormone (AMH) is an important marker of ovarian reserve and has been proposed as an alternative criterion for the diagnosis of PCOS. This study verifies whether AMH and body mass index (BMI) values are predictors of pregnancy in infertile women with PCOS undergoing artificial insemination (AI), a less invasive and painless technique of assisted reproductive technologies (ART).

**Methods:**

This retrospective observational study involved 220 women with PCOS who underwent AI between 2010 and 2022. Participants were categorized into three groups based on BMI and serum AMH levels. To categorize the three AMH classes, the 25th (4.08ng/mL) and 75th (8.99ng/mL) AMH percentiles were defined as cut-offs, and the words ‘low’, ‘middle’, and ‘high’ were utilized to define the groups.

**Results:**

There was a tendency towards a decrease in reproductive outcomes (number of inseminations with positive human-chorionic gonadotropin, number of live births, and number of term births) with an increase in the BMI value. All of these outcomes were also slightly higher in women with ‘middle’ AMH levels compared to women with ‘low’ and ‘high’ AMH. However, none of these results were statistically significant.

**Conclusions:**

This study suggests BMI may be an important predictive factor for pregnancy and there appears to be a range of biological normality for AMH values, where ‘low’ and ‘high’ levels of this hormone could constitute a marker of poor reproductive prognosis, in women with PCOS undergoing AI.

## INTRODUCTION

Polycystic ovarian syndrome (PCOS) is the most common endocrine condition in women of reproductive age, affecting them from adolescence to menopause (Gao *et al*., 2022; Teede *et al*., 2023). It has a prevalence of 8-13% and accounts for almost 80% of cases of anovulatory infertility (Gao *et al*., 2022; Sivanandy & Ha, 2023; Teede *et al*., 2023). This syndrome is characterized by an increase in the pulsatility of gonadotropin-releasing hormone (GnRH), which stimulates the secretion of luteinizing hormone (LH), which in turn increases the production of androgens by theca cells, resulting in a high luteinizing hormone/follicle-stimulating hormone (LH/FSH) ratio (Sadeghi *et al*., 2022; Teede *et al*., 2023). Women with PCOS have a diverse clinical presentation, including gynecological/reproductive, metabolic, endocrine, and psychiatric features (Gao *et al*., 2022; Rudnicka *et al*., 2021). In terms of gynecology, these women may experience oligomenorrhea, amenorrhea, and/or infertility (di Clemente *et al*., 2022; Gao *et al*., 2022; Teede *et al*., 2023). Hirsutism, acne, and alopecia are endocrine symptoms and indicators of clinical hyperandrogenism (di Clemente *et al*., 2022; Gao *et al*., 2022; Teede *et al*., 2023). Regardless of age or body mass index (BMI) at the time of diagnosis, PCOS increases the risk of cardiovascular disease, high blood pressure, dyslipidemia, type 2 diabetes mellitus, obesity, metabolic dysfunction-associated steatotic liver disease (MASLD), and obstructive sleep apnea (di Clemente *et al*., 2022; Gao *et al*., 2022; Teede *et al*., 2023). Women with PCOS are more likely to develop depression and anxiety disorders, as well as endometrial hyperplasia and cancer (Teede *et al*., 2023). All of these problems worsen the patients’ infertility. Furthermore, pregnant women with this syndrome are at a higher risk of miscarriage, gestational diabetes, hypertension, fetal growth restriction, preterm birth, and cesarean section (Gao *et al*., 2022).

This pathology is diagnosed using the 2003 Rotterdam criteria (Rotterdam ESHRE/ASRM-Sponsored PCOS consensus workshop group, 2004), which require the presence of two of the following three criteria: (1) oligoor anovulation, (2) clinical/laboratory hyperandrogenism, and (3) ultrasound-detected polycystic ovaries (Sivanandy & Ha, 2023). Before making a clear diagnosis of PCOS, other endocrine illnesses (thyroid dysfunction, hyperprolactinemia, congenital adrenal hyperplasia, Cushing’s syndrome, among others) must be ruled out (Collée *et al*., 2021). Regarding criterion (1), the definition of oligomenorrhea differs according to the woman’s reproductive stage (Teede *et al*., 2023). Thus, oligomenorrhea is physiological around menarche and menopause; if it occurs 1-3 years after menarche, it is defined as menstrual cycles longer than 45 days; if it occurs 3 years or more after menarche, it is defined as cycles longer than 35 days or when women have fewer than 8 normal-length cycles per year (Gao *et al*., 2022; Teede *et al*., 2023). Regarding criterion (2), hyperandrogenism can appear as hirsutism or acne; however, when these signs are mild or absent, a biochemical examination can be performed by measuring total and free testosterone levels (Gao *et al*., 2022). Finally, criterion (3) requires using ultrasound to detect 20 or more follicles in at least one ovary, with the transvaginal technique being the most accurate (Gao *et al*., 2022; Teede *et al*., 2023).

Anti-Müllerian hormone (AMH) is a peptide produced by the granulosa cells of pre-antral and small antral ovarian follicles from the 36th week post-conception until menopause, and it is an essential marker of ovarian reserve (di Clemente *et al*., 2022; Rudnicka *et al*., 2021; Teede *et al*., 2023). AMH levels can be tested in both serum and follicular fluid and vary with age and BMI (Gao *et al*., 2022; Moreira *et al*., 2023; Teede *et al*., 2023). After a brief perinatal surge, AMH levels stay low until they peak at puberty and then gradually decline throughout reproductive life until menopause (di Clemente *et al*., 2022; Rudnicka *et al*., 2021; Sadeghi *et al*., 2022; Sivanandy & Ha, 2023). Furthermore, serum AMH appears to vary among women with a higher BMI (Gao *et al*., 2022), and the link between these two factors is not consistent between studies. In women with regular menstrual cycles, AMH values are lower in the luteal phase compared to the follicular phase. However, this variation is not significant and AMH can be measured on any day, unlike FSH and LH, which must be assessed at the beginning of the follicular phase (Teede *et al*., 2023). AMH regulates sexual differentiation by suppressing the development of Müllerian ducts in male fetuses (Rudnicka *et al*., 2021; Teede *et al*., 2023). The absence of AMH in female fetuses allows Müllerian ducts to mature and give rise to the fallopian tubes, uterus, cervix, and upper part of the vagina (Teede *et al*., 2023). This hormone also controls ovarian folliculogenesis by choosing only one dominant follicle and limiting the recruitment of other primordial follicles via a paracrine mechanism (Rudnicka *et al*., 2021). AMH is also important for raising LH secretion and androgen synthesis while decreasing expression of the LH receptor and aromatase, an enzyme that converts androgens into estrogens (di Clemente *et al*., 2022; Rudnicka *et al*., 2021).

Serum AMH levels in women with PCOS are 2 to 4 times greater than in women without PCOS, owing to an increased number of pre-antral follicles and small antral follicles, as well as AMH upregulation by granulosa cells (di Clemente *et al*., 2022). This explains the high LH/FSH ratio and the large levels of androgens in these women, and also the possibility of a positive feedback loop between AMH, LH, and GnRH in PCOS (Rudnicka *et al*., 2021). Because of the close relationship between serum AMH levels and preantral and antral ovarian follicle counts on ultrasound, AMH has been advocated as an alternative diagnostic criterion, particularly in circumstances when ultrasound examination of the ovaries is challenging (Rudnicka *et al*., 2021). Recent research shows that replacing criterion (3) of the Rotterdam criteria with serum AMH levels can effectively predict the existence of PCOS with a high sensitivity (78 to 100%) and specificity (88 to 100%) (Rudnicka *et al*., 2021; Teede *et al*., 2023). The most commonly proposed AMH cohort point in Caucasians is 4.9 ng/mL (Rudnicka *et al*., 2021), however other research has already attempted to create alternative cut-offs for the ideal AMH value according to the outcomes of IVF techniques in these patients facing infertility (Vale-Fernandes *et al*., 2023a).

The initial step in treating PCOS in women is to lose weight and limit calorie intake by regular exercise and a fatand sugar-free diet (Sadeghi *et al*., 2022). A 5-10% weight loss can normalize the menstrual cycle and restore fertility (Collée *et al*., 2021; Sadeghi *et al*., 2022). In the second line, medicines such as combined oral contraceptives, antidiabetics (metformin, liraglutide, orlistat), and anti-androgens (spironolactone, finasteride) can be administered alone or in combination, depending on the patient’s goals (Gao *et al*., 2022; Sadeghi *et al*., 2022). Bariatric surgery might be viewed as the final stage in weight loss (Gao *et al*., 2022).

If weight loss alone is ineffective for infertility treatment, ovulation induction is used, with letrozole or clomiphene citrate as first-line medications (Gao *et al*., 2022). In the second phase, gonadotropins or ovarian drilling are recommended, and in the final phase, assisted reproductive technologies (ART) (Gao *et al*., 2022). In vitro fertilization (IVF) with single embryo transfer was the primary technique used; however, in recent years, some clinical trials have focused on a less invasive, less expensive, and painless technique, artificial insemination (AI) or intrauterine insemination (IUI), and have shown that it can be used as a first step before proceeding to IVF (Sivanandy & Ha, 2023). Approximately 98% of pregnancies occurred in the first three AI cycles (Sivanandy & Ha, 2023).

AI is an ART approach that allows sperm to cross natural barriers in the female vaginal canal (Allahbadia, 2017; Bayer *et al*., 2018). AI can be conducted with or without prior ovarian stimulation, and in the vast majority of cases, this is done to boost the technique’s success rate (Allahbadia, 2017). After ovulation is triggered, a vaginal ultrasound is done on the 12th day of the menstrual cycle to monitor the size of the ovarian follicles (Bayer *et al*., 2018). When the follicle reaches 18 mm, recombinant-human chorionic gonadotropin (r-HCG) is given, and insemination is scheduled 36 hours later (Bayer *et al*., 2018). On the day of insemination, a semen sample is collected and then cleaned and processed to remove bacteria and prostaglandins while also concentrating the sperm (Bayer *et al*., 2018). AI is performed using a speculum, which facilitates the insertion of a catheter into the uterine cavity and allows prepared sperm to flow through, followed by a pregnancy test 14 days later (Bayer *et al*., 2018).

The aim of this study is to determine if AMH and BMI levels are predictors of conception in PCOS-infertile women undergoing AI.

## MATERIALS AND METHODS

### Scientific research

The electronic databases Pubmed, ScienceDirect, and SciELO were searched for relevant studies using the search terms “PCOS” or “Polycystic Ovary Syndrome” in combination with “Intrauterine Insemination” or “IUI”, “artificial inseminations” or “AI”, “Assisted Reproductive Technologies” or “ART”, “Body Mass Index” or “BMI”, and “Anti-Müllerian Hormone” or “AMH”. Before reading the articles in full, the title and abstract were used to choose them, with no language restrictions.

### Study design

This is a retrospective and observational study carried out at the Center for Medically Assisted Procreation (CPMA, in Portuguese), a public ART center, of the Centro Materno- Infantil do Norte Dr. Albino Aroso (CMIN) and approved by the Ethics Committee of the Unidade Local de Saúde de Santo António / Institute of Biomedical Sciences Abel Salazar (ULSSA/ICBAS), Porto, Portugal (Reference - 2023.278[239-DEFI/230-CE]).

### Selection criteria

All women diagnosed with PCOS using the Rotterdam criteria (Rotterdam ESHRE/ASRM-Sponsored PCOS consensus workshop group, 2004) and undergoing AI between January 1, 2010, and December 31, 2022, were included, for a total of 220 women with PCOS and 359 AI cycles. In all AI cycles conducted at our center none of the clinical cases presented any male factor infertility - all individuals presented a normal sperm count in the initial evaluation according to the WHO criteria (Cooper *et al*., 2010). At our center, AI is only performed on women who have one to two good-sized follicles during stimulation; otherwise, if a greater number of follicles are observed, the cycle is canceled, and contraception is recommended, as we do not convert these cycles to IVF due to specific access rules and a waiting list for IVF treatments at the public centers. Additionally, while few women have only one patent fallopian tube, if this occurs and stimulation is detected on that side, the cycle is canceled. Intervention was ovarian stimulation and/or AI if post wash total motile sperm count was > 8 million (Evans *et al*., 2020). Mature follicles were defined as ≥ 14 mm as measured on the day of ovulation trigger (Evans *et al*., 2020). Exclusion criteria included donor treatments and a probable diagnosis of PCOS without matching the Rotterdam criteria, as well as the presence of clinical/biochemical hyperandrogenism caused by other factors than PCOS. The data (age, BMI, FSH, LH, AMH, number of inseminations, pregnancies with positive beta-human chorionic gonadotropin (β-HCG), full-term pregnancies, and miscarriages) were taken from the CMIN CPMA’s anonymized database. The study participants were divided into three groups based on age, BMI, and serum AMH levels. The following age groups were considered: class 1 (<31 years), class 2 (31-40 years), and class 3 (>40 years). BMI classification: class 1 (<25 kg/m^2^), class 2 (25-30 kg/m^2^), and class 3 (>30 kg/m^2^). To categorize the three AMH classes, the 25th (4.08 ng/mL) and 75th (8.99 ng/mL) AMH percentiles were defined as cut-offs, and the words ‘low’, ‘middle’, and ‘high’ were utilized. Therefore, the ‘low’ group comprises the AI cycles of women with AMH levels below the 25th percentile (n=29), the ‘middle’ group comprises the AI cycles of women with AMH levels between the 25th and 75th percentile (n=58) and the ‘high’ group comprises AI cycles of women with AMH levels above the 75th percentile (n=29). These classification categories ‘low’, ‘middle’, and ‘high’ are used only to simplify the description of the results and allow for better comparison, so they are not synonyms for biological normality.

### Outcomes

The key outcome is the number of AI-induced pregnancies and live deliveries, as well as their relationship to BMI and serum AMH levels. Pregnancies were confirmed by testing serum β-HCG levels around 14 days after insemination. Secondary outcomes include the number of miscarriages and full-term deliveries.

### Statistical analysis

Statistical analysis was carried out using the Statistical Package for the Social Sciences version 29 (IBM SPSS 29) software. Continuous variables were described as mean±standard deviation, whereas categorical variables were represented by the number of cases and percentages. Pearson’s Chi-square test was employed to evaluate significance for categorical variables, while the ANOVA test was used to check for variance homogeneity in continuous data. If the result was negative, the Kruskal-Wallis test was applied. Pearson’s correlation was used to determine the degree of relationship between reproductive outcomes in women with PCOS who underwent AI and BMI and serum AMH levels. A linear correlation test was also run between the AMH classes, BMI, and age. A *p*-value <0.05 indicated statistical significance result.

## RESULTS

A sample of 220 PCOS women who received a total of 359 AI cycles was examined. These cycles produced 75 pregnancies, which equates to 20.89%. The sample included women aged 21-41 years, with an average age of 31.45 years (±4.24) and a BMI of 25.86 kg/m^2^ (± 5.21). The average FSH and LH levels were 6.17 (± 1.68) mIU/mL and 8.90 (± 4.60) mIU/mL, respectively, with an LH/FSH ratio of 1.48 (± 0.69). The average blood AMH level among these women was 7.30 ng/mL (± 5.10). Table 1 describes the characteristics of the women in the sample.

**Table 1:** Clinical characteristics of women with Polycystic Ovary Syndrome (PCOS) undergoing artificial insemination (AI).

	Mean±SD	Minimum	Maximum
**Age (years)**	31.45±4.24	21	41
**BMI (Kg/m^2^)**	25.86±5.21	17	43
**FSH (mIU/mL)**	6.17±1.68	2.3	15.8
**LH (mIU/mL)**	8.90±4.60	2.3	38
**LH/FSH ratio**	1.48±0.69	0.39	5.1
**AMH (ng/mL)**	7.30±5.10	1.31	29.52

BMI: body mass index; FSH: follicle-stimulating hormone; LH: luteinizing hormone; AMH: anti-Müllerian hormone; IU: international units; SD: standard deviation.

The women in the sample (n=220) were split based on their BMI and serum AMH levels. In terms of BMI (Table 2), no statistically significant differences were found between the traits and reproductive outcomes examined and BMI. However, there was a trend towards fewer conceptions, live births, and term births when the BMI increased.

**Table 2:** Reproductive characteristics, hormonal levels and reproductive outcomes in artificial insemination (AI) cycles according to body mass index (BMI) values.

	Normal BMI (n=110)	Overweight (n=62)	Obesity (n=42)	p-value
Age (years)	31.45±4.18	31.40±4.00	31.52±4.82	NS^a^
FSH (mIU/mL)	6.23±1.50	6.43±1.83	5.67±1.83	NS^a^
LH (mIU/mL)	8.66±4.09	10.08±5.91	7.85±3.71	NS^a^
LH/FSH ratio	1.42±0.67	1.60±0.80	1.43±0.54	NS^a^
Positive β-HCG [n (%)]	31 (46.3)	23 (34.3)	13 (19.4)	NS^b^
Live birth [n (%)]	23 (44.2)	19 (36.5)	10 (19.2)	NS^b^
Miscarriage [n (%)]	5 (62.5)	3 (37.5)	0 (0)	NS^b^
Term birth [n (%)]	20 (46.5)	16 (37.2)	7 (16.3)	NS^b^

Values expressed as mean±standard deviation (SD). *p*-value < 0.05 was considered statistically significant. BMI: body mass index; FSH: follicle-stimulating hormone; LH: luteinizing hormone; β-HCG: beta-human chorionic gonadotropin; IU: international units; NS: not statistically significant.

a ANOVA test.

b Chi-square test.

The sample’s age, BMI, and serum AMH values did not follow a normal distribution. 84.5% (n=186) of the women were under 31 years old, whereas 50% (n=110) had a normal body weight (BMI<25 kg/m^2^).

Despite 47.2% (n=104) AMH missing results, 13.2% (n=29) were below the 25th AMH percentile (AMH≤4.08 ng/mL), 26.4% (n=58) between the 25th and 75th AMH percentile, and 13.2% (n=29) above the 75th AMH percentile (AMH≥8.99 ng/mL).

In this regard, a correlation study was performed, revealing a significant negative association between BMI and FSH (r=-0.152; *p*=0.031). The remaining associations were not statistically significant (Table 3).

**Table 3:** Correlation between body mass index (BMI) and the characteristics in artificial insemination (AI) cycles and the outcomes studied. The asterisk (^*^) denotes statistically significant values.

	BMI r (Pearson’s Correlation Coefficient)	p-value
Age	0.007	NS
FSH (mIU/mL)	-0.152	0.031^*^
LH (mIU/mL)	-0.036	NS
LH/FSH ratio	0.036	NS
No. of positives β-HCG	0.042	NS
No. of live births	0.063	NS
No. of miscarriages	-0.052	NS

*p*-value<0.05 was considered statistically significant. BMI: body mass index; FSH: follicle-stimulating hormone; LH: luteinizing hormone; β-HCG: beta-human chorionic gonadotropin; IU: international units; NS: not statistically significant.

Table 4 shows that women with PCOS and ‘low’ serum AMH levels are older and have a lower BMI, but this is not statistically significant (*p*>0.05). However, women with ‘high’ AMH levels are younger (*p*=0.018), and have a lower FSH value (*p*=0.026), a higher LH value (‘middle’ *vs*. ‘high’: *p*=0.018; ‘low’ *vs*. ‘high’: *p*=0.025) and, subsequently, a higher LH/FSH ratio (‘middle’ *vs*. ‘high’: *p*=0.006; ‘low’ *vs*. ‘high’: *p*<0.001). Women with ‘middle’ AMH levels had somewhat greater reproductive outcomes (number of AIs with positive β-HCG, number of live births, number of miscarriages, and number of term births) than those with ‘low’ or ‘high’ AMH levels, but there were no statistically significant differences.

**Table 4:** Reproductive characteristics, hormonal levels and reproductive outcomes in artificial insemination (AI) cycles according to anti-Müllerian hormone (AMH) values.

	‘Low’ AMH (<4.08ng/mL) (n=29)	‘Middle’ AMH (4.08-8.99ng/mL) (n=58)	‘High’ AMH (>8.99ng/mL) (n=29)	p-value
**Age (years)**	32.76±3.48	31.17±4.75	29.79±4.09	0.018^a,c^
**BMI (kg/m^2^)**	24.53±3.38	25.95±5.37	27.12±5.67	NS^a^
**FSH (mIU/mL)**	6.97±1.33	6.27±1.60	6.07±1.67	0.026^a,c^
**LH (mIU/mL)**	9.09±6.16	8.67±3.19	13.25±7.49	0.025^a,c^, 0.018^a,d^
**LH/FSH ratio**	1.29±0.73	1.40±0.41	2.13±1.06	<0.001^a,c^, 0.006^a,d^
**Positive β-HCG [n (%)]**	10 (27)	19 (51.4)	8 (21.6)	NS^b^
**Live birth [n (%)]**	8 (29.6)	12 (44.4)	7 (25.9)	NS^b^
**Miscarriage [n (%)]**	1 (25)	4 (80)	0 (0)	NS^b^
**Term birth [n (%)]**	7 (30.4)	10 (43.5)	6 (26.1)	NS^b^

Values expressed as mean±standard deviation (SD). *p*-value < 0.05 was considered statistically significant. AMH: anti-Müllerian hormone; BMI: body mass index; FSH: follicle-stimulating hormone; LH: luteinizing hormone; β-HCG: beta-human chorionic gonadotropin; IU: international units; NS: not statistically significant.

a ANOVA test complemented with Kruskal-Wallis test.

b Chi-square test.

c Low-high comparison.

d Middle-high comparison.

Furthermore, there was a significant positive relationship between serum AMH levels, serum LH levels, and the LH/FSH ratio (Table 5). On the other hand, there was an inverse relationship found between blood AMH levels and age and serum FSH levels. There were no significant differences found between serum AMH levels and BMI (r=0.126; *p*=0.177), the number of AI cycles with positive β-HCG (r=0.026; *p*=0.783), the number of births alive (r=0.049; *p*=0.598), or the number of miscarriages (r=-0.061; *p*=0.515).

**Table 5:** Correlation between anti-Müllerian hormone (AMH) and the characteristics in artificial insemination (AI) cycles and the outcomes studied. The asterisk (^*^) denotes statistically significant values.

	AMH r (Pearson's Correlation Coeficient)	p-value
**Age**	-0.248	0.007^*^
**BMI**	0.126	NS
**FSH (mIU/mL)**	-0.218	0.025^*^
**LH (mIU/mL)**	0.323	<0.001^*^
**LH/FSH ratio**	0.483	<0.001^*^
**No. of positives β-HCG**	0.026	NS
**No. of live births**	0.049	NS
**No. of miscarriages**	-0.061	NS

*p*-value < 0.05 was considered statistically significant. AMH: anti-Müllerian hormone; BMI: body mass index; FSH: follicle-stimulating hormone; LH: luteinizing hormone; β-HCG: beta-human chorionic gonadotropin; IU: international units; NS: not statistically significant.

To rule out potential confounding factors, a linear regression analysis was conducted between the number of AI cycles with positive β-HCG and age, BMI, and AMH value, yielding a *p*-value of 0.876.

## DISCUSSION

The World Health Organization (WHO) recommends that ART be used as a last resort for treating infertility in women with PCOS (Gao *et al*., 2022). While the effectiveness of different ART methods has been debated, AI is increasingly being used as a first step before IVF. AI is considered less invasive, more affordable, and painless (Sivanandy & Ha, 2023), even though IVF typically leads to better reproductive outcomes (Bayer *et al*., 2018). However, there is limited research on how BMI and AMH levels affect the success of AI in women with PCOS, especially in cases where ovulatory issues are present but sperm counts are normal.

Women in this study had an average BMI over 25 kg/m^2^, indicating they were above the normal weight range. Additionally, they exhibited high serum levels of LH and AMH, along with an increased LH/FSH ratio. These findings are consistent with existing literature on the physical and hormonal characteristics of PCOS women (Sadeghi *et al*., 2022; Teede *et al*., 2023). To understand the role of BMI in shaping these hormonal changes, we examined findings from other studies. Research indicates that there are significant differences in FSH, LH, and the LH/FSH ratio between obese and non-obese women (Lal *et al*., 2017). Specifically, a notable negative correlation exists between BMI and FSH levels in our results as previously documented by another research group (Kiddy *et al*., 1990). However, while some studies have found that overweight or obese women have lower LH levels (Chen *et al*., 2018), our study did not find this association. Similarly, we observed no significant relationship between BMI and the LH/FSH ratio, which aligns with previous research (Alnakash & Al-Tae’e, 2007; Saadia, 2020). The relationship between BMI, LH, FSH, the LH/FSH ratio, and AMH in women with PCOS is complex. While there may be some influence of BMI on hormonal levels, the inconsistencies across studies highlight the need for more research. Future studies should focus on understanding the factors that contribute to these relationships, such as insulin resistance and inflammation, to improve treatment options for managing PCOS.

BMI has been identified as a significant predictor of pregnancy outcomes, both in natural conception and following ART (Aydin *et al*., 2013; Chen *et al*., 2018). Our investigation observed a trend indicating a decline in the number of pregnancies as BMI increased; however, this trend did not reach statistical significance. This finding aligns with the results of previous studies that have reported similar outcomes (Guan *et al*., 2021; Sheng *et al*., 2017; Souter *et al*., 2011). Moreover, our results corroborate existing literature that indicates no significant differences in live birth rates or miscarriage rates associated with varying BMI levels (Guan *et al*., 2021; Sheng *et al*., 2017; Souter *et al*., 2011).These findings contribute to the broader understanding of how BMI may modulate reproductive outcomes, suggesting that other factors may play a more crucial role in influencing pregnancy success in this population.

AMH presents distinct advantages over other female hormones in reproductive assessments, as its levels remain stable throughout the menstrual cycle, exhibit minimal variability between cycles, and are less influenced by the operator’s skill in measurement (Elgindy *et al*., 2008). Consequently, evaluating serum AMH levels may serve as a reliable reference for counseling women with PCOS regarding the prognosis of pregnancy outcomes following AI (Li *et al*., 2010). Our investigation revealed a significant inverse correlation between AMH levels and age, which aligns with existing literature documenting the gradual decline of AMH due to ovarian aging and subsequent deterioration of oocyte quality (Bertone-Johnson *et al*., 2018; de Kat *et al*., 2016; Dondik *et al*., 2017; Gunasheela *et al*., 2021; Kruszyńska A, Słowińska-Srzednicka, 2017; Li *et al*., 2010; Tal *et al*., 2014). This decline in AMH is typically associated with an increase in serum FSH levels, confirming the findings of both our study and earlier research (Bhattacharya *et al*., 2022; Desforges-Bullet *et al*., 2010; Dewailly *et al*., 2020; Guo *et al*., 2021; Homburg *et al*., 2013; Li *et al*., 2010; Tal *et al*., 2014; Xi *et al*., 2012). Conversely, our study indicated a positive association between serum AMH and LH levels, which is corroborated by previous investigations (Bhattacharya *et al*., 2022; Desforges-Bullet *et al*., 2010; Dewailly *et al*., 2020; Guo *et al*., 2021; Homburg *et al*., 2013; Li *et al*., 2010; Lin *et al*., 2011; Piouka *et al*., 2009; Tal *et al*., 2014; Vale-Fernandes *et al*., 2023a; Xi *et al*., 2012). Additionally, a positive correlation was identified between AMH levels and the LH/FSH ratio. While this ratio is commonly utilized in the diagnosis of PCOS (Le *et al*., 2019; Tola *et al*., 2018), its prognostic value for pregnancy outcomes in women undergoing AI remains inadequately explored. Regarding the relationship between BMI and serum AMH levels, our study found a non-significant trend suggesting that women with lower AMH levels tended to have lower BMI. However, this association is not universally acknowledged, as some studies report a positive correlation (Dondik *et al*., 2017), while others indicate a negative correlation (Cui *et al*., 2014; Gao *et al*., 2022; Kriseman *et al*., 2015; Vagios *et al*., 2021; Zhang *et al*., 2023). The variability in findings may be influenced by differences in the age groups studied, suggesting that age could act as a confounding variable in the relationship between AMH and BMI. Overall, these findings underscore the importance of AMH as a potential biomarker for predicting reproductive outcomes in women with PCOS undergoing ART, particularly in the context of AI. Further research is essential to clarify the implications of AMH levels in this population and to explore potential confounding factors that may influence these relationships.

AMH is widely recognized as a key biomarker for assessing ovarian reserve (di Clemente *et al*., 2022; Rudnicka *et al*., 2021; Teede *et al*., 2023), and elevated levels of AMH may correlate with an increased risk of adverse reproductive outcomes (Vale-Fernandes *et al*., 2023a; Vale-Fernandes *et al*., 2024). In our analysis, while we observed no significant differences, women classified with ‘low’ and ‘high’ AMH levels demonstrated slightly decreased rates of inseminations resulting in positive β-HCG when compared to women with ‘middle’ AMH levels. In a study involving 360 infertile women from France without a diagnosis of PCOS, pregnancy rates following a single cycle of AI were evaluated based on AMH percentiles. Specifically, participants were stratified according to the 20th and 80th percentiles of AMH, which correspond to approximately 1 ng/mL and 4.5 ng/mL, respectively. The authors reported no statistically significant differences in pregnancy rates among the various AMH groups (Lamazou *et al*., 2012). Conversely, another study indicated that women with serum AMH levels equal to or exceeding 2.3 ng/mL experienced significantly higher pregnancy rates compared to those with lower AMH levels (Moro *et al*., 2016). Furthermore, our data revealed that women with ‘middle’ AMH levels experienced a marginally higher number of live births and miscarriages; however, these differences were not statistically significant. This observation contrasts with findings from other studies suggesting that higher AMH concentrations may be associated with an improved likelihood of achieving a live birth (Stalzer *et al*., 2023). In the context of preterm birth, existing literature indicates an elevated risk in women with PCOS (Boomsma *et al*., 2006; Kjerulff *et al*., 2011; Qin *et al*., 2013); however, our investigation found no association between AMH levels and the incidence of term deliveries. These results align with prior research that suggests a biological spectrum of normalcy: ‘low’ AMH levels are indicative of diminished ovarian reserve, while ‘high’ AMH levels may be associated with increased disease severity and poorer reproductive prognosis (Guo *et al*., 2021; La Marca *et al*., 2004; Lin *et al*., 2011; Piouka *et al*., 2009; Sun *et al*., 2021; Tal *et al*., 2020; Tal *et al*., 2014; Vale-Fernandes *et al*., 2023a). One possible explanation for these findings is that elevated AMH concentrations may lead to aberrant folliculogenesis, alterations in endometrial receptivity, and placental dysfunction (Tal *et al*., 2020; Vale-Fernandes *et al*., 2024). Overall, our findings contribute to the complex understanding of AMH as a prognostic biomarker in reproductive outcomes for women undergoing AI, particularly those affected by PCOS. Further longitudinal studies are warranted to elucidate the nuanced relationships between AMH levels, ovarian reserve, and reproductive outcomes across diverse populations.

Despite the established biological reference range for AMH levels, numerous studies have endeavored to define specific cut-off values for both the diagnosis of PCOS and the assessment of reproductive prognosis. Our research group has previously reported proposed cut-off thresholds for healthy women that may enhance ovarian response following gamete donation (Oliveira *et al*., 2023; Vale-Fernandes *et al*., 2023b) and for infertile women undergoing IVF treatments to predict poor and high response to stimulation (Reis *et al*., 2024). In the context of IVF, several studies have identified cut-off levels of AMH that optimize its diagnostic utility in women with PCOS. For instance, a study conducted in India established a cut-off of 3.34 ng/mL, indicating a sensitivity of 98% and a specificity of 93% for predicting ovarian response (Saikumar *et al*., 2013). Additionally, another investigation determined that an AMH cut-off of 4.9 ng/mL exhibited the strongest correlation between sensitivity and specificity (Wiweko *et al*., 2014). While there has been increasing research into the diagnostic and prognostic roles of AMH in the context of IVF cycles for women with PCOS, there remains a significant gap in knowledge regarding its implications for AI. The present study represents a pioneering effort to explore the relationship between serum AMH levels and reproductive outcomes in women with PCOS undergoing AI. Given the importance of AMH as a potential biomarker in reproductive medicine, further investigation is warranted to elucidate its prognostic value in this specific setting.

This study contains several limits and strengths. The use of percentiles rather than absolute AMH scores improves the generalizability of the findings. The study’s biggest limitation is its retrospective nature, which means that the analysis is based on previously collected data, making it impossible to acquire more information on specific variables. Furthermore, AMH levels were only routinely obtained starting in 2017, limiting the sample size on this parameter (there is 47.27% missing AMH values - out of the total sample of 220 participants, AMH values were documented and assessed in 116 women). Another limitation is that the study was conducted in a single public center with a predominantly Caucasian population, failing to account for racial/ethnic differences in AMH levels, as well as variations caused by genetic and environmental factors such as obesity, smoking, and vitamin D deficiency (Iliodromiti *et al*., 2013; Tal & Seifer, 2013). This study was conducted in a public center where AI treatments are restricted to women under the age of 42, and IVF is limited to those under 40. Consequently, the sample population is confined to a relatively narrow age range, which constrains the ability to perform stratified analyses based on chronological age. The study did not address the differences between having one or two growing follicles, which could introduce selection analyses bias and covered both natural and stimulated insemination cycles, but did not individually analyze the various ovulation-inducing drugs. Several trials, however, have demonstrated that stimulation with clomiphene citrate, letrozole, and gonadotropins was equally effective and safe in promoting conception in women with PCOS (Huang *et al*., 2018). Although all men met the minimum insemination criteria mentioned in the methodology, a comparative analysis based on sperm profile was not carried out, which constitutes a limitation to consider. All individuals presented a normal sperm count in the initial evaluation according to the WHO criteria (Cooper *et al*., 2010) and according some authors AI is a well-supported first-line treatment for moderate male factor subfertility and is considered cost-effective (Ombelet *et al*., 2003). However, identifying specific semen parameters that predict pregnancy outcomes after AI is challenging due to inconsistencies in semen analysis standards and other influencing factors, such as patient selection and ovarian stimulation methods (Ombelet *et al*., 2003). While research indicates that strict sperm morphology criteria and inseminating motile sperm count are critical for AI success, a definitive threshold for optimal semen quality remains undetermined, although rates appear to decline with fewer than 5% normal sperm and an inseminating motile sperm count below 1 million (Ombelet *et al*., 2003). A significant limitation of the present study pertains to the nature of the data that can be analyzed within the context of AI cycles. These cycles represent first-line techniques in ART, characterized by their relatively straightforward and minimally invasive nature. Consequently, specific details regarding oocyte and embryonic characteristics remain unknown. Nevertheless, it is important to note that this study encompasses a substantial cohort of treatments involving women diagnosed with PCOS undergoing AI. The findings yield valuable insights into the reproductive prognosis for this population, which often exhibits a poor reproductive outlook even in the context of IVF treatments (Vale-Fernandes *et al*., 2023a).

## CONCLUSION

While this study did not find statistically significant results, it suggests a trend where higher BMI is linked to fewer pregnancies in infertile women with PCOS undergoing AI. This supports the idea that BMI could be an important predictor of pregnancy outcomes, highlighting the need for continued weight loss efforts in this group.

This study further elucidates women with ‘low’ and ‘high’ AMH levels exhibit reduced rates of conception and live births, although these findings did not achieve statistical significance. It appears that there exists a median range of biological normality concerning AMH levels. Consequently, one may infer that both ‘low’ and ‘high’ AMH levels may correlate with an unfavorable reproductive prognosis in women with PCOS undergoing AI, consistent with our previous research findings in the context of IVF (Vale-Fernandes *et al*., 2023a).
